# m^6^A Reader HNRNPA2B1 Promotes Esophageal Cancer Progression via Up-Regulation of ACLY and ACC1

**DOI:** 10.3389/fonc.2020.553045

**Published:** 2020-09-29

**Authors:** Huimin Guo, Bei Wang, Kaiyue Xu, Ling Nie, Yao Fu, Zhangding Wang, Qiang Wang, Shouyu Wang, Xiaoping Zou

**Affiliations:** ^1^Department of Gastroenterology, The Affiliated Drum Tower Hospital of Nanjing University Medical School, Nanjing, China; ^2^Yancheng First Hospital, Affiliated Hospital of Nanjing University Medical School, Yancheng, China; ^3^Department of Radiotherapy, The First Affiliated Hospital of Nanjing Medical University, Nanjing, China; ^4^Department of Pathology, The Affiliated Drum Tower Hospital of Nanjing University Medical School, Nanjing, China; ^5^Department of Hepatobiliary Surgery, The Affiliated Drum Tower Hospital of Nanjing University Medical School, Nanjing, China; ^6^Jiangsu Key Laboratory of Molecular Medicine, Medical School of Nanjing University, Nanjing, China; ^7^Center for Public Health Research, Medical School of Nanjing University, Nanjing, China

**Keywords:** m^6^A, esophageal cancer, HNRNPA2B1, fatty acid synthesis, ACLY, ACC1

## Abstract

N6-methyladenosine (m^6^A) modification is the most abundant modification on eukaryotic RNA. In recent years, lots of studies have reported that m^6^A modification and m^6^A RNA methylation regulators were involved in cancer progression. However, the m^6^A level and its regulators in esophageal cancer (ESCA) remain poorly understood. In this study, we analyzed the expression of m^6^A regulators using The Cancer Genome Atlas data and found 14 of 19 m^6^A regulators are significantly increased in ESCA samples. Then we performed a univariate Cox regression analysis and LASSO (least absolute shrinkage and selection operator) Cox regression model to investigate the prognostic role of m^6^A regulators in ESCA, and the results indicated that a two-gene prognostic signature including ALKBH5 and HNRNPA2B1 could predict overall survival of ESCA patients. Moreover, HNRNPA2B1 is higher expressed in high-risk scores subtype of ESCA, indicating that HNRNPA2B1 may be involved in ESCA development. Subsequently, we confirmed that the level of m^6^A and HNRNPA2B1 was significantly increased in ESCA. We also found that HNRNPA2B1 expression positively correlated with tumor diameter and lymphatic metastasis of ESCA. Moreover, functional study showed that knockdown of HNRNPA2B1 inhibited the proliferation, migration, and invasion of ESCA. Mechanistically, we found that knockdown of HNRNPA2B1 inhibited the expression of *de novo* fatty acid synthetic enzymes, ACLY and ACC1, and subsequently suppressed cellular lipid accumulation. In conclusion, our study provides critical clues to understand the role of m^6^A and its regulators in ESCA. Moreover, HNRNPA2B1 functions as an oncogenic factor in promoting ESCA progression via up-regulation of fatty acid synthesis enzymes ACLY and ACC1, and it may be a promising prognostic biomarker and therapeutic target for human ESCA.

## Introduction

Esophageal cancer (ESCA) is one of the major malignant cancers that threatened human health worldwide (1, 2). Esophageal squamous cell carcinoma (ESCC) and esophageal adenocarcinoma (EAC) are the two common subtypes of ESCA, especially the ESCC accounting for 80% in China (3). Over the last several decades, improved treatments have prolonged the survival of ESCA patients diagnosed at an early stage; however, most ESCA patients are first diagnosed at an advanced stage with malignant proliferation and metastasis (4). Surgical resection combined with radiotherapy and chemotherapy has improved the prognosis of ESCA patients, but the overall 5-year survival rate remains extremely poor (5). Therefore, identifying novel biomarkers and therapeutic targets for ESCA patients is an urgent need.

It is well-known that lots of chemical modifications on human RNA were involved in the development of human diseases, including cancer (6, 7). Recent studies reveal that N6-methyladenosine (m^6^A) modification is the most abundant modification involved in the progression of different cancers (6, 8–10). The m^6^A modification accounts for ~0.1–0.4% of adenosine on isolated mammals RNA (6, 11). The level of m^6^A is reversible and dynamic, which could be installed by m^6^A methyltransferases (writers) or removed by m^6^A demethylases (erasers). In addition, the specific RNA-binding proteins (readers) could recognize and bind to m^6^A motif, regulating RNA metabolism, including RNA stability, degradation, splicing, transport, localization, translation, and others (9, 12). Lots of studies have shown that the writers, erasers, and readers are closely associated with the characteristics of cancer, including tumor proliferation, apoptosis, metastasis, angiogenesis, drug resistance, energy metabolism, and cancer stem cell (6, 8, 13–16). Despite the function of m^6^A modification and its regulators in different malignant cancers have been reported, its role in ESCA has not been studied so far.

In the present study, we systematically analyzed the expression of 19 m^6^A RNA regulators in ESCA using The Cancer Genome Atlas (TCGA) dataset, as well as their association with the clinicopathological characteristics. After a comprehensive analysis, we found that HNRNPA2B1 may play a key role in ESCA development. Subsequently, we found that the levels of m^6^A and its regulator HNRNPA2B1 were significantly increased in ESCA, and HNRNPA2B1 acts an oncogenic role in the progression of ESCA cells, indicating that it may be a promising prognostic biomarker and therapeutic target for human ESCA.

## Materials and Methods

### Selection of m^6^A RNA Methylation Regulators

Previous studies have reported the bioinformatics analysis of total 13 m^6^A-related genes in gastric cancer (17), bladder cancer (18), and other cancers (19, 20). In our study, a total of 19 m^6^A RNA methylation regulators were included for systematically analysis based on the recent the m^6^A review (9).

### Bioinformatics Analysis

The RNA-seq transcriptome data and clinical information of ESCA patients were obtained from TCGA (https://cancergenome.nih.gov/). All 19-gene expression data are downloaded via the R package “TCGA-Assembler.” The expression of 19 m^6^A-related genes in 160 ESCA tissues and 11 normal esophageal tissues was analyzed via limma package. Next, the violin map was used to visualize the expression of 19 genes in 160 ESCA tissues and 11 normal tissues. We then used the STRING database (http://string-db.org) to analyze the protein–protein interaction (PPI) among 19 m^6^A regulators. The correlation analysis of 19 m^6^A regulators was further analyzed by Pearson correlation analysis. To evaluate the association between the expression of m^6^A regulators and prognosis of ESCA patients, the ESCA cohort was clustered into different groups through consensus clustering analysis with “ConsensusClusterPlus” in R (21). The overall survival (OS) difference was calculated by the Kaplan–Meier method and log-rank test. A χ^2^-test was used to compare the distribution of age, gender, grade, and stage between different clusters. Univariate Cox analysis was performed to evaluate the correlation between m^6^A regulators and OS of ESCA patients using survival analysis in R. Two m^6^A genes were selected for further analysis, and a risk signature was developed using the least absolute shrinkage and selection operator (LASSO) Cox regression algorithm. The formula of the risk score for ESCA patients' prognosis prediction was as follows: risk score = the sum of each multivariate cox regression coefficient ratio of mRNA multiple each expression of mRNA. Based on the median risk score, we divided the patients into high- and low-risk subgroups. Each patient's survival status, death time, and gene expression profile in two subgroups were presented via “heatmap” and “survival” R packages (22). Besides, the Kaplan–Meier curve analysis was performed, and the receiver operating characteristic (ROC) curve was drawn to estimate the sensitivity and specificity of the prognostic signature. The ESCA cohort was divided into high- and low-risk group based on the median value of the risk scores. OS between different clusters or groups was calculated by the Kaplan–Meier method. ROC curve was constructed to evaluate the prediction accuracy of ESCA prognosis (22). The distribution of clinicopathological parameters between high- and low-risk group was analyzed through χ^2^-test. Univariate and multivariate Cox regression analyses were used to identify the independent prognostic factors.

### ESCC Tissue, Tissue Microarray, and Cell Culture

Eighteen pathologically confirmed ESCC tissues from recent patients at the Nanjing Drum Tower Hospital, the Affiliated Hospital of Nanjing University Medical School (Nanjing, Jiangsu, China), were obtained after signed informed consent. The ESCC tissue microarray (TMA, 34 cases) was obtained from servicebio (Wuhan, Hubei, China). Immunohistochemistry (IHC) was performed according to standard procedures as described previously (8, 23). Institutional approval was obtained from the Review Board of Nanjing Drum Tower Hospital prior to this study. Human esophageal epithelial cell line HEEpiC and ESCC cell lines (ECA109 and TE10) were purchased from the Type Culture Collection of the Chinese Academy of Sciences (Shanghai, China). All cells were cryopreserved at −80°C using CELLSAVING (C40100, New Cell & Molecular Biotech, China). All cell lines were cultured in RPMI-1640 medium supplemented with 100 μg/mL streptomycin, 100 U/mL penicillin, and 10% fetal bovine serum (FBS). Oleate (OA) was obtained from Sigma.

### siRNA Constructs and Transfection

The two specific HNRNPA2B1 siRNAs were designed and synthesized by RiboBio (Guangzhou, China): the sequence of si-HNRNPA2B1#1: GGAGAGTAGTTGAGCCAAA and the sequence of si-HNRNPA2B1#2: GCTACGGAGGTGGTTATGA. The HNRNPA2B1 siRNAs and corresponding control siRNA were transfected into the ESCC cells by DharmaFECT4 (Dharmacon, Chicago, IL).

### Dot Blot Assay

The dot blot assay was performed according to the bio-protocol database (https://en.bio-protocol.org/e2095). The experiments procedures have been described previously (8).

### Western Blot Assay

Western blot assays were performed as previously reported (23). The following antibodies were used: anti-GAPDH (1:2,000; Beyotime, Shanghai, China) and anti-HNRNPA2B1 (1:1,000, Proteintech Group, Rosemont, IL, USA). Phenylmethylsulfonyl fluoride used in Western blot assay was from Selleck (Houston, TX, USA).

### Quantitative Real-Time Reverse Transcription–Polymerase Chain Reaction Assay

Total RNA was extracted from cells or tissues using TRIzol reagent (Invitrogen, Carlsbad, CA, USA). Reverse transcription (RT) was performed with HiScript Q RT SuperMix for qPCR (Vazyme, Jiangsu, China). RT–polymerase chain reaction (PCR) was performed with an SYBR Green PCR Kit (Vazyme, Jiangsu, China) on an Applied Biosystems 7900HT sequence detection system (Applied Biosystems), with triplicate reactions. The primers used are listed in Supplementary Table 1.

### Proliferation Assay

For CCK8 assay, after the cells were transfected for 48 h, the cells were plated at a density of 2,000 cells per well in 96-well-plates. After 72 h, cell viability was determined using CCK-8 assay according to the manufacturer's instructions (APExBIO, Houston, TX, USA).

For clonogenic assay, after the cells were transfected for 48 h, the cells were seeded at a density of 500 cells per well in 12-well-plates and incubated at 37°C for 10–14 days. Then, cells were fixed with methanol for 30 min and stained with crystal violet (Beyotime, Shanghai, China) for 30 min.

For EdU assay, the cells were plated at a density of 10,000 cells per well in 96-well-plates, and the cells were transfected for 48 h. The cell immunofluorescence was determined by the EdU kit according to the manufacturer's instructions (RiboBio, Guangzhou, China). Images of the cells were acquired with a Leica DMi8 system.

### Cell Mobility Assay

For wound-healing assay, the transfected cells were grown to confluence in a 12-well-plate. Next, the cells were cultured in RPMI-1640 with 1% FBS for 12 h. The confluent monolayer was then disrupted with a cell scraper and filmed at the indicated hours via Leica DMi8 system. The rate of wound closure was calculated as the ratio of the average distance between the two wound edges and the total cell duration of migration.

For Transwell assay, the transfected cells in 100 μL of serum-free medium were seeded in the upper chambers coated with or without 50 μL of Matrigel (BD Biosciences), and 600 μL of culture medium containing 10% FBS was placed in the lower chambers. After 12 h of incubation at 37°C, cells that migrated to the bottom of the membrane were fixed with 4% paraformaldehyde for 30 min, stained with crystal violet (Beyotime, Shanghai, China) for 30 min, and imaged.

### Nile Red Staining

The transfected cells were fixed in 4% paraformaldehyde solution on the 12-well-plates, stained with 0.05 μg/mL Nile red (Sigma, USA) for 10 min, washed with phosphate-buffered saline twice, and then stained with DAPI (Beyotime, Shanghai, China). Images of the cells were acquired with a Leica DMi8 system.

### Statistical Analyses

The expression of 19 m^6^A-related genes in ESCA tissues and normal tissues from TCGA dataset was analyzed via one-way analysis of variance. Kolmogorov–Smirnov test was used to analyze the relationship between m^6^A-related genes and clinicopathological characteristics. The median risk score was used as a cutoff value to divide into a high- and a low-risk group. OS was analyzed between different groups through the Kaplan–Meier method. The relationship between the risk score and clinicopathological variables was analyzed through χ^2^-test. Functional experiments were performed at least three times. The representative data shown are means ± SD; *P* < 0.05 was considered statistically significant.

## Results

### The Expression and Correlation of m^6^A RNA Methylation Regulators in ESCA

**Figure 1:**
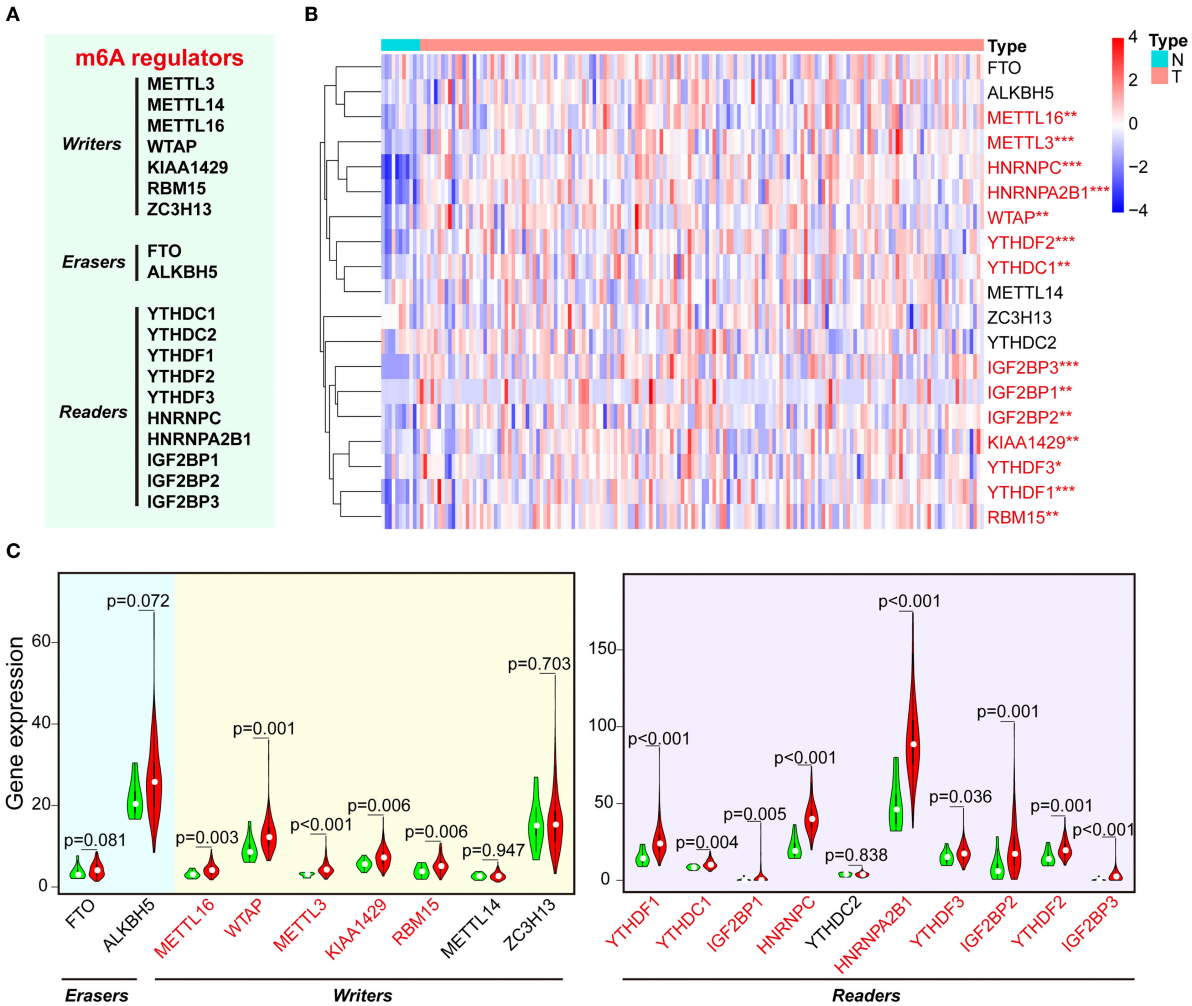
The expression of m^6^A-related regulators in ESCA and normal control samples of TCGA. **(A)** The list of m^6^A RNA methylation–related writers, erasers, and readers. **(B)** The expression levels of 19 m^6^A-related genes in ESCA were displayed via heatmap. N, non-tumor tissues; T, tumor tissues. **(C)** Vioplot visualizing the expression of m^6^A-related genes in ESCA. Green color represents non-tumor tissues, and the red color represents tumor tissues. *P < 0.05, **P < 0.01, and ***P < 0.001.

First, we analyzed the level of 19 m^6^A RNA methylation regulators (7 writers, 2 erasers, and 10 readers) in ESCA tissues and normal tissues from TCGA dataset (Figure 1A). We found that 14 of 19 m^6^A-related genes were significantly increased in ESCA tissues compared with the normal tissues through heatmap visualization (Figure 1B). The expression levels of five writers (METTL16, WTAP, METTL3, KIAA1429, and RBM15) and nine readers (YTHDF1/2/3, YTHDC1, IGF2BP1/2/3, HNRNPC, and HNRNPA2B1) were significantly up-regulated in ESCA tissues, whereas no significant difference was found for the two erasers (FTO and ALKBH5) (Figure 1C). We further analyzed the interaction among the 19 m^6^A-related genes using PPI network, and KIAA1429 and METTL14 seemed to be the center in the interaction network (Supplementary Figure 1A). Using Pearson correlation analysis, we found the correlation between 19 m^6^A-related genes was weak, and it was shown that KIAA1429 is most correlated with YTHDF3 (Supplementary Figure 1B).

### Identification of Prognostic Signature Among m^6^A Regulators in ESCA

Next, the consistent clustering analysis was carried out based on the expression similarity of m^6^A-related genes in ESCA from TCGA dataset. The *k* = 2 seemed to be the most appropriated selection to cluster the patients into two subgroups (cluster 1 and cluster 2) (Supplementary Figures 2A–D). Moreover, we analyzed the clustering result and clinical outcomes, and the results showed that cluster 1 subgroup had a shorter OS than cluster 2 subgroup, although it showed a borderline statistical significance (*P* = 0.064) (Supplementary Figure 2E).

**Figure 2:**
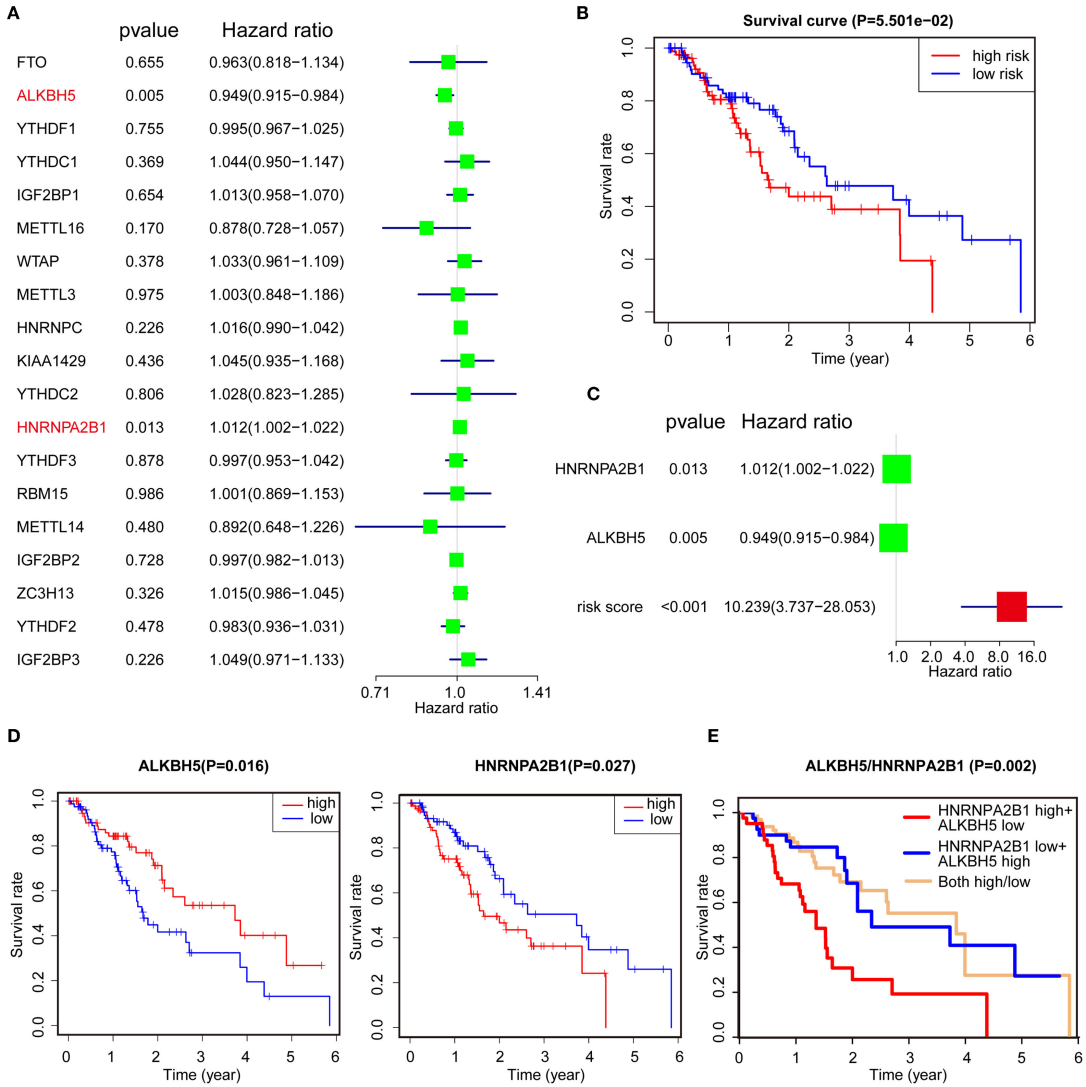
Identification of prognostic signature among m^6^A regulators. **(A)** Identification of the m^6^A-related genes that significantly correlated with OS via univariate analysis. **(B)** Kaplan–Meier OS curves for ESCA patients assigned to high- and low-risk groups. **(C)** The risk signature of ALKBH5 + HNRNPA2B1 is more reliable for OS prognosis via univariate analysis. **(D,E)** Kaplan–Meier curves depicting OS according to the expression ALKBH5 or HNRNPA2B1 **(D)** and combined with ALKBH5/HNRNPA2B1 **(E)** based on TCGA data.

To better understand the role of the 19 m^6^A regulators in the prognosis of ESCA patients, univariate Cox regression was used to analyze the expression of m^6^A-related genes associated with OS in ESCA TCGA dataset. The results demonstrated that high expression of ALKBH5 was significantly correlated with good OS [*P* = 0.005, hazard ratio (HR) = 0.949, 95% confidence interval (CI) = 0.915–0.984], but high expression of HNRNPA2B1 was associated with poor OS (*P* = 0.013, HR = 1.012, 95% CI = 1.002–1.022) (Figure 2A). Furthermore, we applied the LASSO Cox regression algorithm to establish the risk signature, and two genes (low of ALKBH5 and high of HNRNPA2B1) were selected to build the risk signature according to the minimum criteria and the coefficients (Supplementary Figures 3A,B). Then, we analyzed the correlation of HNRNPA2B1 expression with ALKBH5 via online bioinformatics tool (http://gepia.cancer-pku.cn/). The results showed that the expression of HNRNPA2B1 was significantly positively correlated with the expressions of ALKBH5 (Supplementary Figure 3C). In order to analyze the prognostic role of the two-gene risk signature, the ESCA patients in TCGA dataset were separated into low- and high-risk groups based on the median risk score, and the results indicated that the high-risk group has a worse survival compared to low-risk groups, although it showed a borderline statistical significance (*P* = 0.05501) (Figure 2B). However, the risk score of the signature of ALKBH5 and HNRNPA2B1 was more significantly associated with poor OS (*P* < 0.001, HR = 10.239, 95% CI = 3.737–28.053) than individual ALKBH5 or HNRNPA2B1 by univariate Cox regression, suggesting that the risk signature of them is more reliable for OS prognosis (Figure 2C). Furthermore, we found that patients with low level of ALKBH5 suffer a poor OS (*P* = 0.016), whereas whose with a high level of HNRNPA2B1 suffer a poor OS (*P* = 0.027; Figure 2D). When combining ALKBH5 and HNRNPA2B1 as a new variable, ESCA patients were divided into three subgroups according to the median of each expressed value: high level of ALKBH5 and low level of HNRNPA2B1, low level of ALKBH5 and high level of HNRNPA2B1, and both of high ALKBH5/ HNRNPA2B1 expression and low ALKBH5/ HNRNPA2B1 expression. Kaplan–Meier curves demonstrated that the subgroup of high level of ALKBH5 and low level of HNRNPA2B1 was much more favorable to the OS than the subgroup of low level of ALKBH5 and high level of HNRNPA2B1 (*P* = 0.002; Figure 2E). Moreover, ROC curve was applied to predict the survival rates for ESCA patients using two-gene signature risk scores in different years (Supplementary Figures 3D–H); the results indicated that it has a good predictive efficiency with the area under the ROC curve within 2, 4, or 5 years (Supplementary Figures 3E, G–H), whereas the result did not show robust prediction within 4 and 5 years (Supplementary Figures 3G,H).

### Validation of the Clinical Relevance of Two-Gene Signature

**Figure 3:**
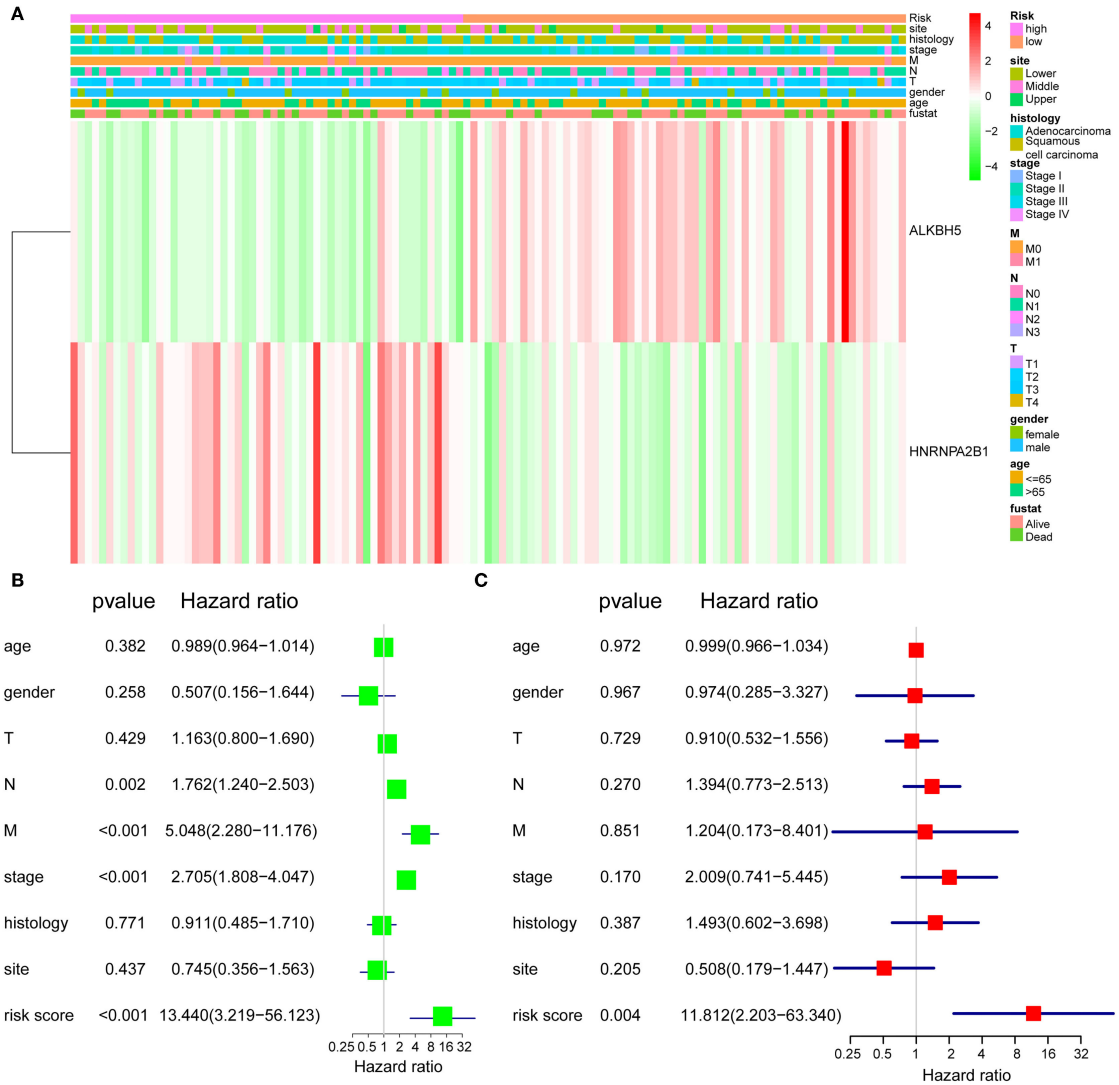
Validation of the prognostic signature. **(A)** The expression levels of the HNRNPA2B1 and ALKBH5 in low- and high-risk ESCA patients. **(B)** Univariate Cox regression analyses of the association between clinicopathological factors and OS of ESCA patients. **(C)** Multivariate Cox regression analyses of the association between clinicopathological factors and OS of ESCA patients.

To better understand the clinical relevance of two-gene signature in ESCA, we first selected the patients with clinical characteristic variables and divided these patients into low- and high-risk groups, which were assessed by two-gene expression and clinical characteristic variables (Figure 3A). Interestingly, we found the most individuals with relative lower expression of ALKBH5 and higher expression of HNRNPA2B1 in the high-risk group, suggesting the risk was associated with the gene expression (Figure 3A). Simultaneously, univariate Cox regression analysis revealed that N stage, stage, and risk score (ALKBH5/HNRNPA2B1 signature) were significantly related with OS of ESCA, and multivariate Cox regression analysis showed that only risk score were an independent prognostic factor for OS of ESCA patients (Figures 3B,C).

### The Correlation Between HNRNPA2B1 and Clinicopathological Features

**Figure 4:**
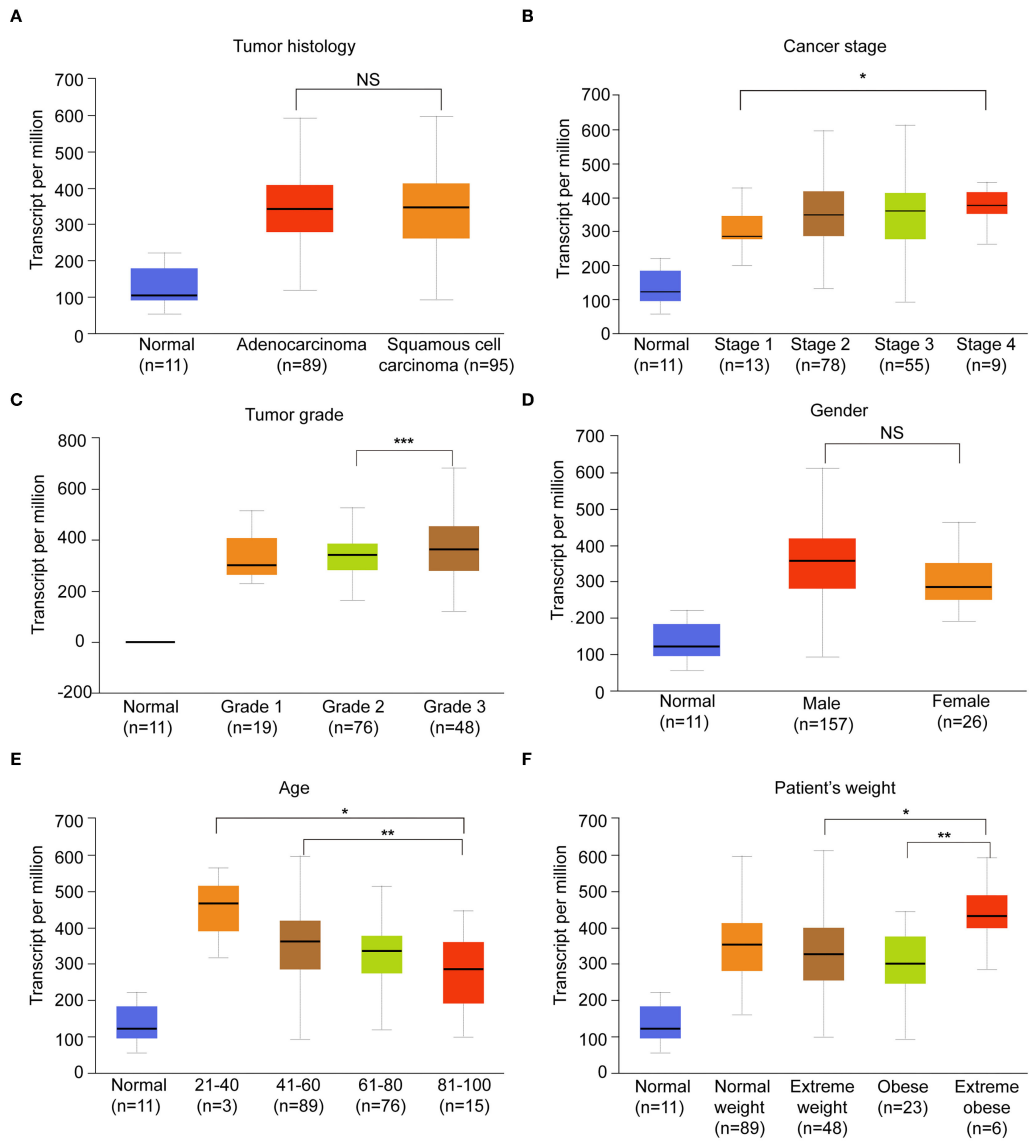
The correlation between HNRNPA2B1 and clinicopathological features. **(A)** The expression of HNRNPA2B1 in ESCA patients of different tumor histology. **(B)** The expression of HNRNPA2B1 in ESCA patients of different cancer stage. **(C)** The expression of HNRNPA2B1 in ESCA patients of different tumor grade. **(D)** The expression of HNRNPA2B1 in ESCA patients of different gender. **(E)** The expression of HNRNPA2B1 in ESCA patients of different age. **(F)** The expression of HNRNPA2B1 in ESCA patients of different weight. *P < 0.05, **P < 0.01, and ***P < 0.001.

Considering that the expression of HNRNPA2B1 is significantly increased in ESCA, while ALKBH5 had no significant difference between ESCA tissues and normal control tissues (Figure 1C), which suggest that HNRNPA2B1 may be involved in ESCA development. We then perform a comprehensive analysis the HNRNPA2B1 expression in different subgroups based on relative clinical characteristics including tumor histology, cancer stage, tumor grade, gender, age, and patient's weight via online bioinformatics tool (http://ualcan.path.uab.edu/index.html). Compared with the normal subgroup, the HNRNPA2B1 expression was significantly up-regulated (*P* < 0.05) in cancer patients with different clinical characteristics (Figures 4A–F). In the ESCA patients, it showed that the HNRNPA2B1 expression between ESCC and EAC had no significant difference (Figure 4A). It also showed that the HNRNPA2B1 expression was not related with gender in ESCA patients (Figure 4D). However, the expression of HNRNPA2B1 significantly increased in the advanced stage and grade (Figures 4B,C). Interestingly, the expression of HNRNPA2B1 was dramatically increased in the young ESCA patients (Figure 4E). Meanwhile, the expression level of HNRNPA2B1 was significantly higher in extreme obese subgroup than other subgroups (Figure 4F), indicating that HNRNPA2B1 may be associated with fatty acid metabolism in ESCA cells.

### The m^6^A Level and HNRNPA2B1 Expression Are Increased in ESCC

**Figure 5:**
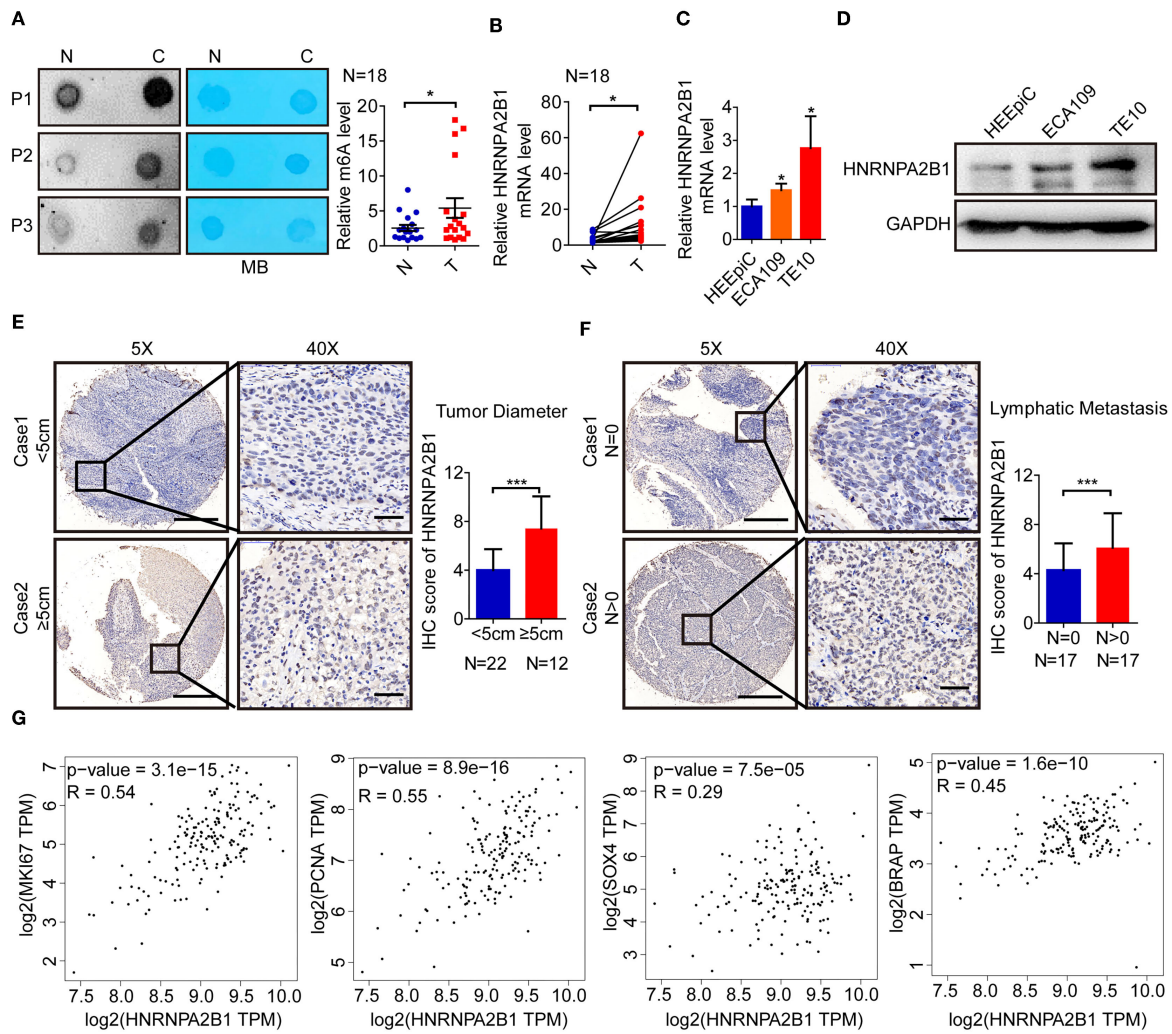
The m^6^A level and HNRNPA2B1 expression are increased in ESCC. **(A)** The mRNAs isolated from ESCC tissues and paired normal tissues were used in dot blot analyses with an anti-m^6^A antibody, and methylene blue (MB) staining served as the loading control (left panel). The relative m^6^A contents on mRNA in ESCC tissues and paired normal tissues were calculated (right panel, *N* = 18). **(B)** The levels of HNRNPA2B1 expression in ESCC and paired normal tissues were measured by qRT-PCR (*N* = 18). **(C)** The levels of HNRNPA2B1 expression in esophageal epithelial cell line (HEEpiC) and ESCC cell lines (ECA109 and TE10) were measured by qRT-PCR. **(D)** The levels of HNRNPA2B1 expression in esophageal epithelial cell line and ESCC cell lines were measured by Western blotting. **(E)** Representative IHC images on the TMA probed with the anti-HNRNPA2B1 antibody (scale bars = 500 or 50 μm, respectively, left panel) are shown. The IHC score of HNRNPA2B1 in ESCC tissues with tumor diameter <5 cm (*N* = 22) and ≥5 cm (*N* = 12) were calculated (right panel). **(F)** Representative IHC images on the TMA probed with the anti-HNRNPA2B1 antibody (scale bars = 500 or 50 μm, respectively, left panel) are shown. The IHC score of HNRNPA2B1 in ESCC tissues with lymphatic metastasis (*N* = 17) or without lymphatic metastasis (*N* = 17) were calculated (right panel). **(G)** The correlation of HNRNPA2B1 expression with the markers of proliferation and metastasis via online bioinformatics tool (http://gepia.cancer-pku.cn/) is analyzed. *P < 0.05, **P < 0.01, and ***P < 0.001.

To elucidate the m^6^A modification in ESCC, we first examined the m^6^A RNA levels in 18 ESCC tissues and paired normal tissues. We found that the m^6^A RNA levels were significantly higher in ESCC tissues via dot blot assay (Figure 5A). Next, we compared the mRNA levels of HNRNPA2B1 in 18 pairs of ESCC and paired normal tissues. The results showed that the mRNA level of HNRNPA2B1 was significantly up-regulated in ESCC (Figure 5B). In addition, the HNRNPA2B1 mRNA and protein level were significantly increased in ESCC cell lines compared with that in normal esophageal epithelial cell lines (Figures 5C,D). To investigate the clinical implication of HNRNPA2B1 with ESCC, we performed IHC staining for HNRNPA2B1 in ESCC TMA. The results indicated that the HNRNPA2B1 level was increased in the tumor diameter of ESCC tissues ≥5 cm compared with that <5 cm (Figure 5E). Similarly, the levels of HNRNPA2B1 protein were also significantly elevated in ESCC tissues with lymph node metastasis than those without lymph node metastasis (Figure 5F). Moreover, we analyzed the correlation of HNRNPA2B1 expression with the markers of proliferation and metastasis via online bioinformatics tool (http://gepia.cancer-pku.cn/). The results showed that the expression of HNRNPA2B1 was significantly positively correlated with the expressions of MKI67 and PCNA, which were the classic biomarkers of proliferative cancer cells (Figure 5G). The expression of HNRNPA2B1 was also significantly positively correlated with SOX4 and BRAP expressions, which were the biomarkers of ESCA metastasis (24, 25) (Figure 5G). Taken together, these results indicated that the levels of m^6^A modification and its regulator HNRNPA2B1 are increased in ESCC, and HNRNPA2B1 may play a critical role in tumor growth and metastasis of ESCC.

### HNRNPA2B1 Promotes ESCC Cell Proliferation

**Figure 6:**
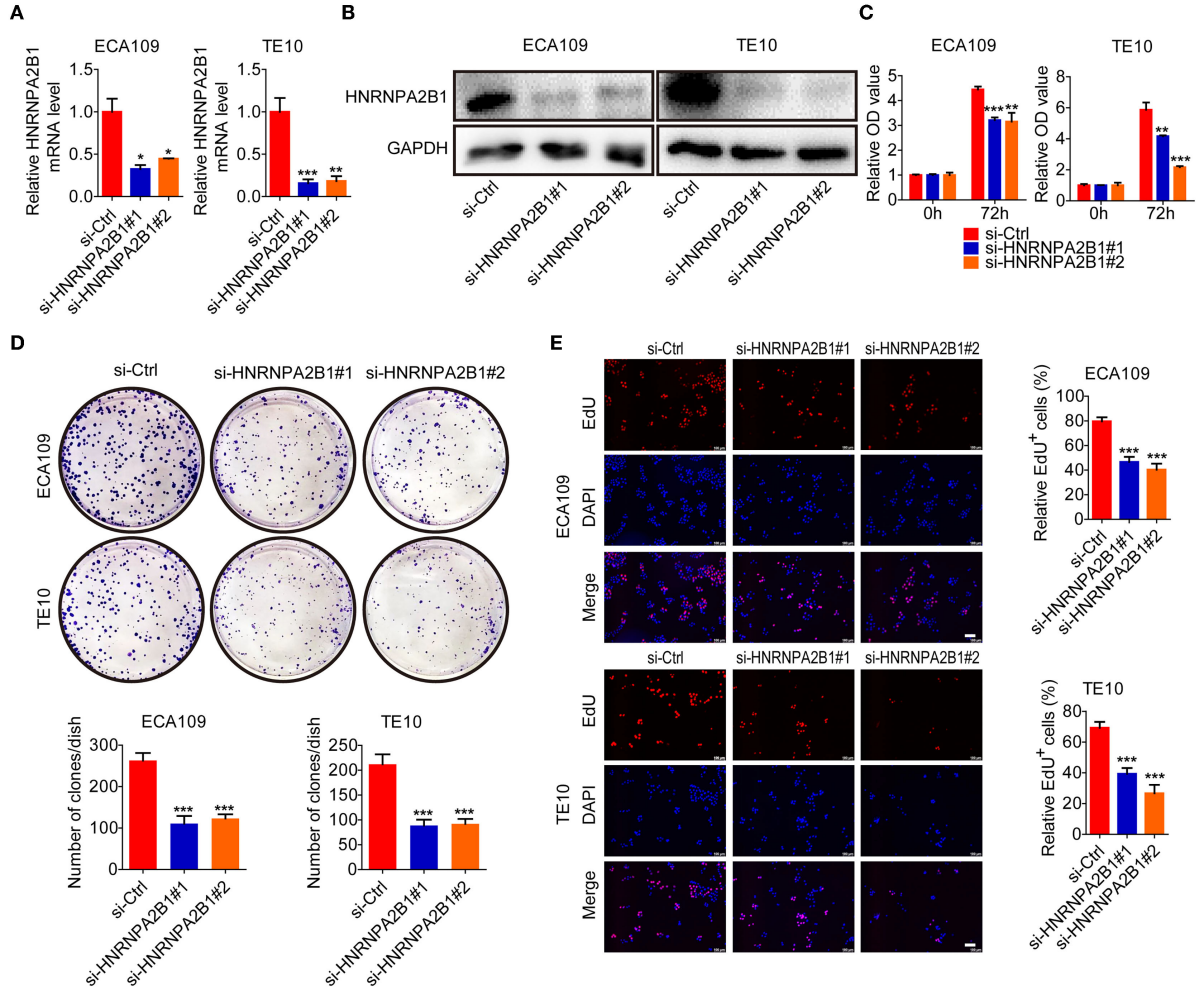
HNRNPA2B1 promotes ESCC cell proliferation. **(A)** The HNRNPA2B1 knockdown efficiency was verified at the mRNA levels in ECA109 and TE10 cells by qRT-PCR assay. **(B)** The HNRNPA2B1 knockdown efficiency was verified at the protein levels in ECA109 and TE10 cells by Western blot assay. **(C)** CCK8 assays showed the growth of ECA109 and TE10 cells upon HNRNPA2B1 knockdown. **(D)** Knockdown of HNRNPA2B1 impaired the colony formation ability of ECA109 and TE10 cells (upper panel). Quantification of the colony formation assay results (bottom panel). **(E)** EdU assays showed the growth of ECA109 and TE10 cells upon HNRNPA2B1 knockdown (left panel). Quantification of the EdU-positive cell results (right panel). Scale bars, 100 μm. *P < 0.05, **P < 0.01, and ***P < 0.001.

To further characterize the role of HNRNPA2B1 in ESCC, we designed and constructed two specific siRNAs to target HNRNPA2B1. The knockdown efficiency was confirmed by qRT-PCR and Western blotting in two ESCC cells (Figures 6A,B). Knockdown of HNRNPA2B1 dramatically suppressed ESCC cell proliferation via CCK8 assay (Figure 6C). As shown in Figure 6D, knockdown of HNRNPA2B1 also significantly inhibited the ESCC cells colony formation. In addition, the EdU assay results also indicated that down-regulation of HNRNPA2B1 could inhibit cell proliferation in ESCC cells (Figure 6E).

### HNRNPA2B1 Promotes ESCC Cell Migration and Invasion

**Figure 7:**
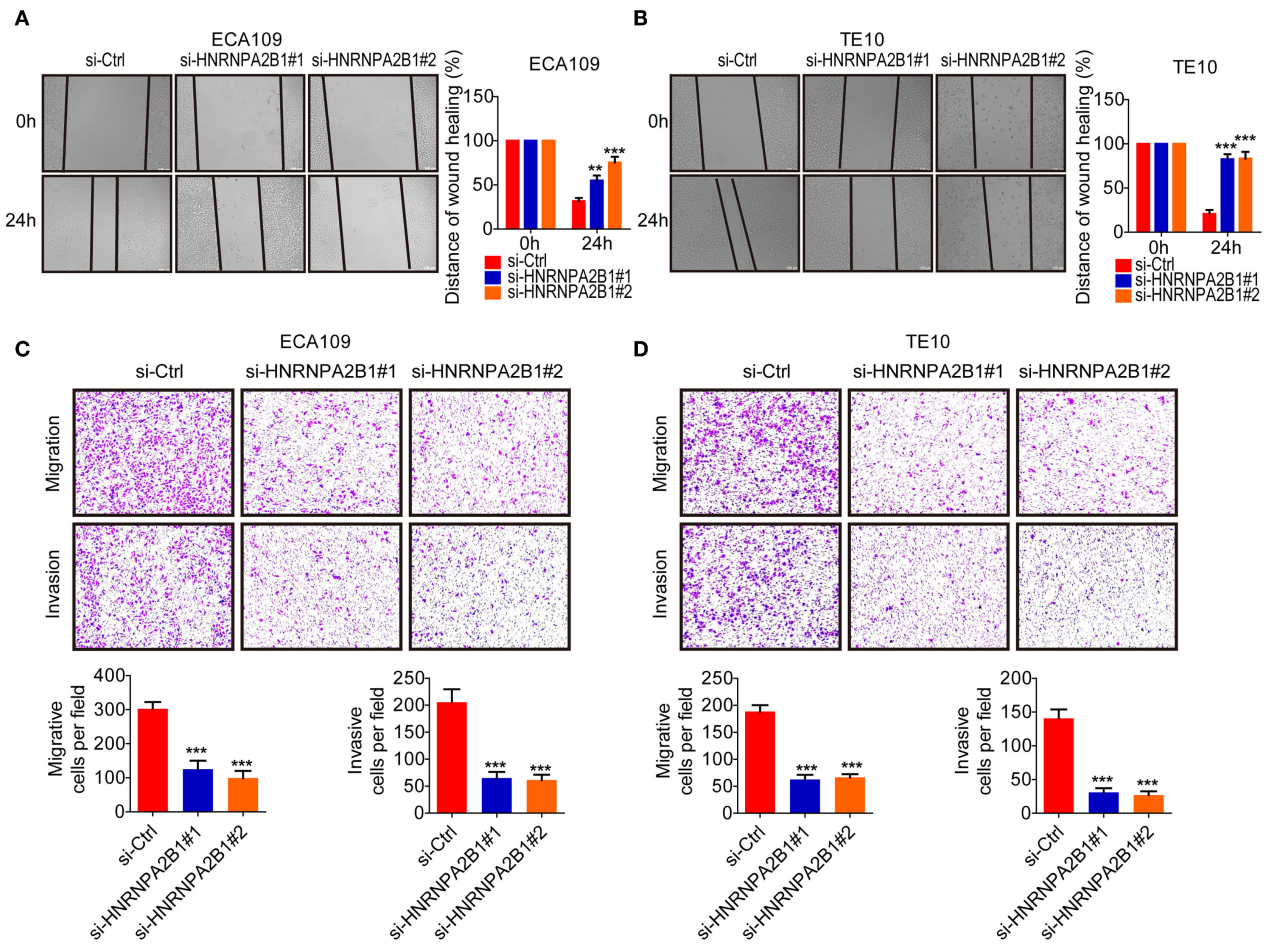
HNRNPA2B1 promotes ESCC cell migration and invasion. **(A,B)** Knockdown of HNRNPA2B1 impaired the migration ability of ECA109 cells **(A)** and TE10 cells **(B)** via wound healing assay (left panel). Quantification of the wound healing assay results (right panel). **(C,D)** Knockdown of HNRNPA2B1 impaired the migration ability of ECA109 cells **(C)** and TE10 cells **(D)** via Transwell assay (upper panel). Quantification of the Transwell assay results (bottom panel). *P < 0.05, **P < 0.01, and ***P < 0.001.

Subsequently, we investigated the role of HNRNPA2B1 in mobility capacity of ESCC cells. The wound healing results showed that knockdown of HNRNPA2B1 suppressed the migration ability of ESCC cells (Figures 7A,B). In addition, the Transwell chamber assays also demonstrated that knockdown of HNRNPA2B1 significantly reduced the migration and invasion of ESCC cells (Figures 7C,D).

### HNRNPA2B1 Accelerates Fatty Acid Synthesis in ESCC

**Figure 8:**
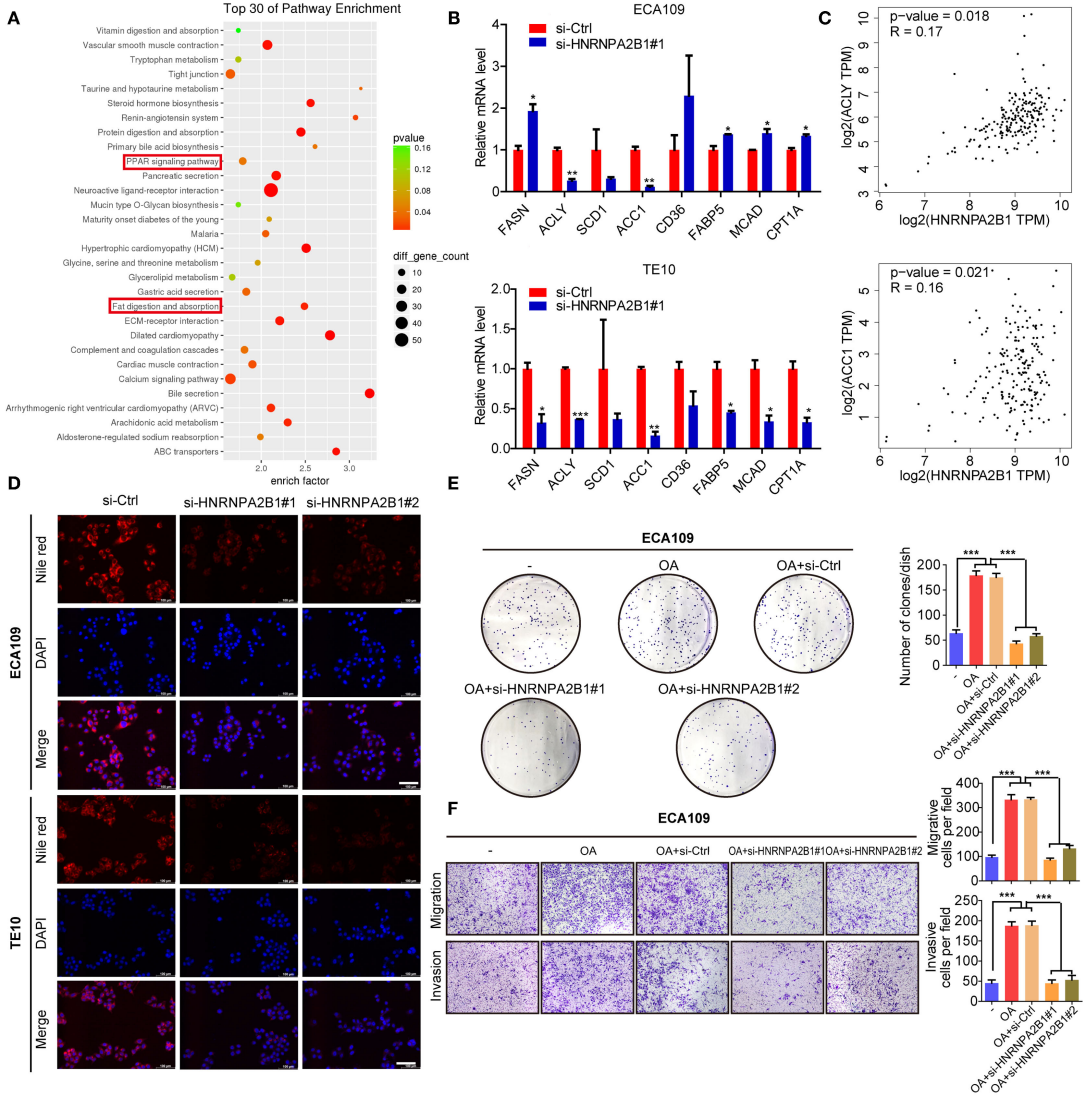
HNRNPA2B1 promotes ESCC progression by regulation of fatty acid metabolism. **(A)** The genes correlated with HNRNPA2B1 expression in ESCA patients were analyzed using TCGA dataset and then the pathway of these genes via KEGG enrichment. **(B)** The mRNA levels of major enzymes in *de novo* fatty acid synthesis (FASN, ACLY, SCD1, and ACC1), fatty acid β-oxidation (MCAD and CPT1A), and fatty acid uptake (CD36 and FABP5) were detected in ECA109 and TE10 cells upon HNRNPA2B1 knockdown. **(C)** The correlations of HNRNPA2B1 expression with ACLY and ACC1 via online bioinformatics tool (http://gepia.cancer-pku.cn/) are analyzed. **(D)** Cellular neutral lipids were measured in ECA109 cells and TE10 cells upon HNRNPA2B1 knockdown by Nile red staining, Scale bars, 100 μm. **(E,F)** OA promoted ESCC cell proliferation, migration, and invasion, whereas knockdown of HNRNPA2B1 inhibited OA-induced ESCC cell proliferation, migration, and invasion. *P < 0.05, **P < 0.01, and ***P < 0.001.

To identify the molecular mechanism involved in HNRNPA2B1 promoting ESCC progression, we first analyzed the genes correlated with HNRNPA2B1 expression in ESCA patients using TCGA data (Supplementary Table 2). Then we analyzed the pathway of these related genes via the Kyoto Encyclopedia of Genes and Genomes (KEGG) enrichment, which showed the pathways included Peroxisome Proliferator-Activated Receptors (PPAR) signaling pathway and fat digestion and absorption (Figure 8A). As shown in Figure 4F, it was suggested that the HNRNPA2B1 level was significantly higher in extreme obese subgroup than other subgroups. Therefore, we investigated whether HNRNPA2B1 could regulate fatty acid metabolism to promote ESCC malignant process. Next, we detected the major enzymes involved in *de novo* fatty acid synthesis, fatty acid β-oxidation and fatty acid uptake, revealing that *de novo* fatty acid synthetic enzymes ACLY, and ACC1 were markedly decreased when knockdown of HNRNPA2B1 in both two ESCC cells (Figure 8B). Moreover, we also found that the expression of HNRNPA2B1 was positively correlated with the expressions of ACLY and ACC1 in ESCA TCGA data via online bioinformatics tool (http://gepia.cancer-pku.cn/, Figure 8C). Meanwhile, knockdown of HNRNPA2B1 suppressed cellular lipid accumulation by staining Nile red in ESCC cells (Figure 8D). Further, we added OA (oleate) into ESCC cells (Supplementary Figure 4A), and the results showed that OA promoted ESCC cell proliferation, migration, and invasion, whereas knockdown of HNRNPA2B1 inhibited OA-induced the malignant process (Figures 8E,F). Collectively, the results reveal that HNRNPA2B1 functions as an oncogenic factor promoting ESCC progression via acceleration of fatty acid synthesis.

## Discussion

ESCA, as a common digestive tract tumor, is a serious threat to human health and contributes to poor prognosis (2, 26). The significant regional difference is the main epidemiological characteristic of ESCA (27). There are two main subtypes in ESCA, called ESCC and EAC, respectively (27, 28). ESCC is mainly in the East Asian population, whereas EAC mainly occurs in Western countries (29). About half of the newly diagnosed ESCA cases in the world occur in China every year (30, 31). ESCA is considered to be a multifactor, multigene, and multistage complicated disease (27). Its occurrence is closely related to chronic nitrosamine stimulation, inflammation and trauma, genetic and epigenetic modification, and other factors (29, 32). Operation, radiotherapy, and chemotherapy are still the main treatment methods for ESCA, but inoperableness and radiochemotherapy resistance limit the clinical effect (29). Therefore, identification of new molecular markers and therapeutic targets is still an urgent need.

The m^6^A modification has become a hot research topic in RNA modification–mediated epigenetic regulation, which was associated with the expression of gene and disease development, including cancer (33, 34). The m^6^A modification is dynamically regulated via the methyltransferases and demethylases (12, 35). Meanwhile, the m^6^A “readers” could recognize m^6^A-modified sites and regulate RNA function. Recent studies have shown that m^6^A modification and its regulators play an important role in various cancers (33). Previous study has systematically characterized the molecular alterations and clinical relevance of 20 m^6^A RNA regulators across 33 cancer types, and they found that m^6^A regulators were found to be potentially useful for prognostic stratification and identified IGF2BP3 as a potential oncogene across multiple cancer types (36). However, the m^6^A level and its regulators in ESCA have not been systematically reported yet. In the present study, we demonstrated that the expression levels of five writers (METTL16, WTAP, METTL3, KIAA1429, and RBM15) and nine readers (YTHDF1/2/3, YTHDC1, IGF2BP1/2/3, HNRNPC, and HNRNPA2B1) were significantly increased in ESCA tissues, whereas no significant difference was found for the two erasers (FTO and ALKBH5), and most of RNA m^6^A regulators in our study were overlapped with the previous study (36). Meanwhile, we analyzed the PPI among 19 m^6^A regulators, which could be systematically and directly helpful to analyze the interaction between these regulators. Herein, we found there are direct or indirect interactions among the 19 m^6^A regulators, and the writers METTL3, METTL14, WTAP, and KIAA1429 and the erasers FTO and ALKBH5 may localized in the center of regulatory network. It was also demonstrated that the relationship between most of the m^6^A RNA methylation regulators is positively correlated, and the KIAA1429 and YTHDF3 genes are most relevant. Subsequently, we confirmed that the RNA m^6^A levels were significantly higher in 18 cancerous tissues than corresponding normal tissues in ESCC patients via a dot blot assay. These results suggest that RNA m^6^A modification may be involved in the ESCA development.

We then analyzed the relationship between RNA m^6^A regulators and OS in ESCA via the consistent clustering analysis, and the results showed that cluster 1 had a shorter OS than cluster 2. In addition, the univariate Cox regression analysis and LASSO Cox regression data indicated that a two-gene prognostic signature including ALKBH5 and HNRNPA2B1 could predict OS of ESCA patients. Moreover, high expression of HNRNPA2B1 and low expression of ALKBH5 were indicated as the risk factor for the survival of ESCA, and the combination of these two factors showed more predictive potential than the alone, although the ROC curve did not show robust prediction within 4 and 5 years, which because of that there are too few patients in the fourth and fifth years, which may lead to the instability of the ROC curve. It is reported that HNRNPA2B1 could selectively bind to m^6^A-containing transcripts via the “m^6^A-switch,” a mechanism in which m^6^A weakens Watson–Crick base pairing to destabilize the RNA hairpin structure and thereby exposes the single stranded hnRNP binding motif (37). HNRNPA2B1 has been reported to be a RNA-binding protein involved in different cancer progression (38–40). HNRNPA2B1 could interact with LINC01234 to promote lung cancer progression (38). It also reported that HNRNPA2B1 promoted malignant capability and inhibited apoptosis via down-regulation of Lin28B expression in ovarian cancer (39). In this study, we also found that HNRNPA2B1 was significantly increased in cancerous tissues of ESCC using TCGA data, which was confirmed in our own samples. Furthermore, we found that HNRNPA2B1 expression positively correlated with tumor diameter and lymphatic metastasis of ESCC. Intriguingly, it was shown that knockdown of HNRNPA2B1 inhibited the proliferation, migration, and invasion of ESCC cell lines, which suggest that HNRNPA2B1 may be critical in the development and progression of ESCA.

Further, we analyzed the KEGG enrichment of genes, which were correlated with HNRNPA2B1 expression in ESCA patients using TCGA data. The data indicated that HNRNPA2B1 may be involved in fatty acid metabolism of ESCA. We also found that the HNRNPA2B1 level was significantly higher in extreme obese subgroup than other subgroups. The abnormal lipid metabolism of tumor cells is mainly manifested in the activation of *de novo* synthesis and oxidative metabolism of fatty acids, which provide the necessary raw materials for tumor cell proliferation (41, 42). The key enzymes related to lipid metabolism play a key role in the abnormal lipid metabolism of tumor cells (43, 44). Subsequently, we detected the expression of major enzymes involved in *de novo* fatty acid synthesis, fatty acid β-oxidation, and fatty acid uptake, revealing that *de novo* fatty acid synthetic enzymes ACLY and ACC1 were markedly positively regulated by HNRNPA2B1. However, the expression of FASN, fatty acid uptake, and fatty acid oxidation genes is inconsistent in the two ESCC cell lines with HNRNPA2B1 deficiency, which may be due to the heterogeneity of the two different ESCC cells. In addition, knockdown of HNRNPA2B1 suppressed cellular lipid accumulation. Collectively, the results reveal that HNRNPA2B1 could accelerate fatty acid synthesis via up-regulation of *de novo* fatty acid synthetic enzymes ACLY and ACC1.

In summary, our findings reveal that the levels of m^6^A and its regulator HNRNPA2B1 were significantly increased in cancerous tissues of ESCA, and overexpression of HNRNPA2B1 promotes ESCA progression via up-regulation of *de novo* fatty acid synthetic enzymes ACLY and ACC1. Therefore, HNRNPA2B1 may be a promising prognostic biomarker and therapeutic target for human ESCA.

## Data Availability Statement

The raw data supporting the conclusions of this article will be made available by the authors, without undue reservation.

## Ethics Statement

This study was approved by the Ethical Committee of Nanjing Drum Tower Hospital. Written informed consent to participate in this study was provided by the patients.

## Author Contributions

SW, QW, and XZ provided the direction of this manuscript. HG, BW, and KX collected and analyzed the data. LN and YF analyzed and evaluated the IHC of TMA. KX and ZW performed the experiments. QW wrote the manuscript. SW revised this manuscript. All authors read and approved the final manuscript.

## Conflict of Interest

The authors declare that the research was conducted in the absence of any commercial or financial relationships that could be construed as a potential conflict of interest.

## Abbreviations

HR: hazard ratio
CI: confidence interval
ESCA: esophageal cancer
m^6^A: N6-methyladenosine
TCGA: The Cancer Genome Atlas
METTL3: methyltransferase-like 3
METTL14: methyltransferase-like 14
METTL16: methyltransferase-like 16
WTAP: WT1 associated protein
KIAA1429, VIRMA: vir like m^6^A methyltransferase associated
RBM15: RNA binding motif protein 15
ZC3H13: zinc finger CCCH-type containing 13
FTO: FTO alpha-ketoglutarate dependent dioxygenase
ALKBH5: alkB homolog 5, RNA demethylase
YTHDC1/2: YTH domain-containing protein 1/2
YTHDF1/2/3: YTH N6-methyladenosine RNA binding protein 1/2/3
IGF2BP1/2/3: insulin like growth factor 2 mRNA binding protein 1/2/3
HNRNPA2B1: heterogeneous nuclear ribonucleoprotein A2/B1
HNRNPC: heterogeneous nuclear ribonucleoprotein C
OS: overall survival
ESCC: Esophageal squamous cell carcinoma
EAC: esophageal adenocarcinoma
PPI: protein-protein interaction
OS: overall survival
LASSO: least absolute shrinkage and selection operator
ROC: Receiver operating characteristic
TMA: tissue microarray
IHC: Immunohistochemistry
AUC: area under the ROC curve
MKI67: marker of proliferation Ki-67
PCNA: proliferating cell nuclear antigen
SOX4: SRY-box transcription factor 4
BRAP: BRCA1 associated protein
FASN: fatty acid synthase
ACLY: ATP citrate lyase
SCD1: stearoyl-Coenzyme A desaturase 1
ACC1: acetyl-CoA carboxylase alpha
MCAD: acyl-CoA dehydrogenase medium chain
CD36: CD36 molecule
FABP5: fatty acid binding protein 5
KEGG: Kyoto Encyclopedia of Genes and Genomes
qRT-PCR: Quantitative real-time RT-PCR
OA: oleate.
