# Supplementary material for: m6A Reader HNRNPA2B1 Promotes Esophageal Cancer Progression via Up-Regulation of ACLY and ACC1
Source: Front Oncol. 2020 Sep 29;10:553045. doi: 10.3389/fonc.2020.553045 (PMC7550530; doi:10.3389/fonc.2020.553045)
Supplement: Supplementary file 1 [file Table_1.docx]

Supplementary Material

# Supplementary Table 1

**The primer sequences for qRT-PCR**

| Primer names | Sequences (5’-3’) |
| --- | --- |
| GAPDH F | CATGTGGGCCATGAGGTCCACCAC |
| GAPDH R | GGGAAGCTCACTGGCATGGCCTTCC |
| HNRNPA2B1 F | ATTGATGGGAGAGTAGTTGAGCC |
| HNRNPA2B1 R | AATTCCGCCAACAAACAGCTT |
| FASN F | AAGGACCTGTCTAGGTTTGATGC |
| FASN R | TGGCTTCATAGGTGACTTCCA |
| ACLY F | TCGGCCAAGGCAATTTCAGAG |
| ACLY R | CGAGCATACTTGAACCGATTCT |
| SCD1 F | AGAATGGAGGAGATAAGT |
| SCD1 R | TAGCAGAGACATAAGGAT |
| ACC1 F | AATGTCCTTCTCCTCCAA |
| ACC1 R | GAGTGAATGAGTTGTCCAA |
| CD36 F | GGCTGTGACCGGAACTGTG |
| CD36 R | AGGTCTCCAACTGGCATTAGAA |
| FABP5 F | TGAAGGAGCTAGGAGTGGGAA |
| FABP5 R | TGCACCATCTGTAAAGTTGCAG |
| MCAD F | ACAGGGGTTCAGACTGCTATT |
| MCAD R | TCCTCCGTTGGTTATCCACAT |
| CPT1A F | TCCAGTTGGCTTATCGTGGTG |
| CPT1A R | TCCAGAGTCCGATTGATTTTTGC |

Abbreviations: F, Forward; R, Reverse.

**
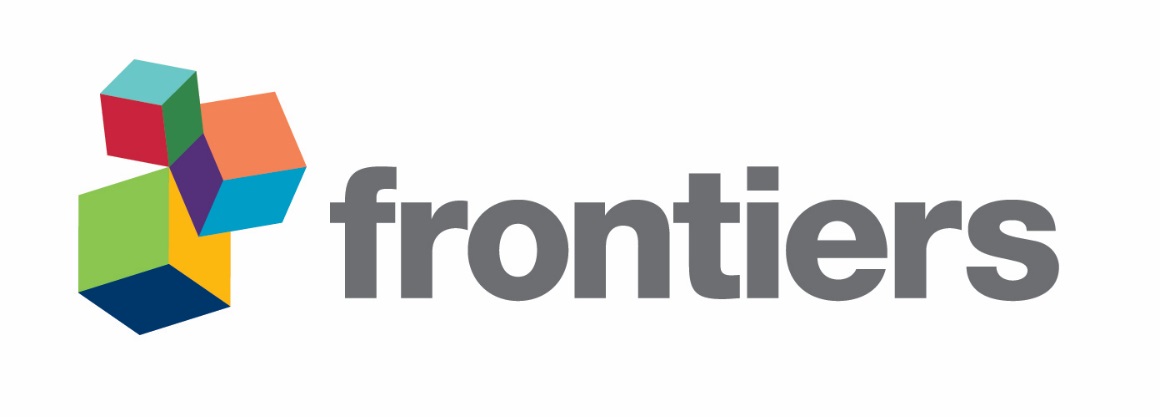
**
