# Supplementary material for: m6A Reader HNRNPA2B1 Promotes Esophageal Cancer Progression via Up-Regulation of ACLY and ACC1
Source: Front Oncol. 2020 Sep 29;10:553045. doi: 10.3389/fonc.2020.553045 (PMC7550530; doi:10.3389/fonc.2020.553045)
Supplement: Supplementary file 3 [file Data_Sheet_2.docx]

Supplementary Material

# Supplementary Figures


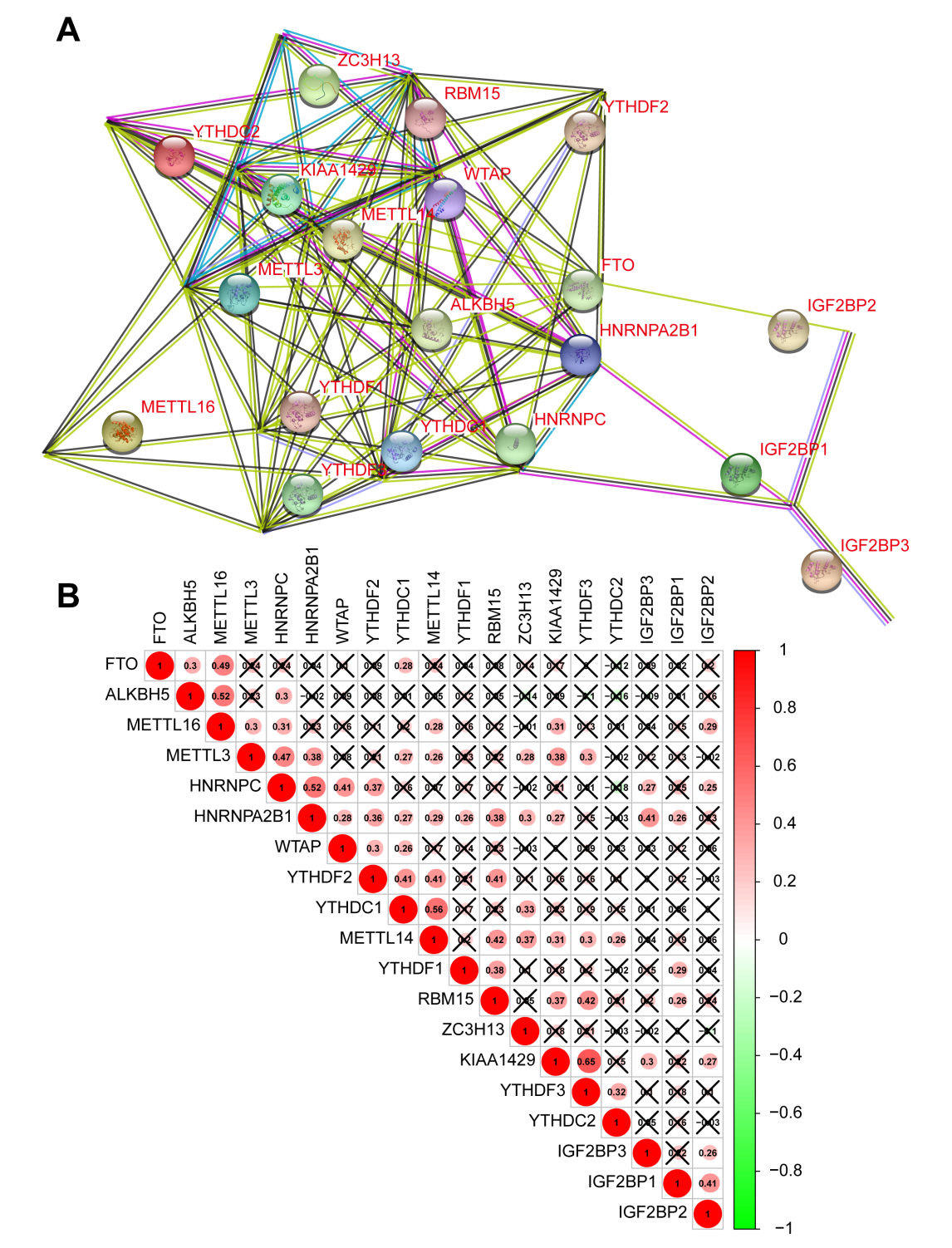


**Supplementary Figure 1. The interaction and correlation of m^6^A related genes.** (A) The interaction among 19 m^6^A genes of ESCA was evaluated via PPI network. (B) The correlation among 19 m^6^A genes of ESCA was evaluated via the Pearson correlation analysis. When P≤0.001, it indicates that there is a correlation among the regulators. Red color represents a positive correlation and green color represents a negative correlation, otherwise, marked with ×.


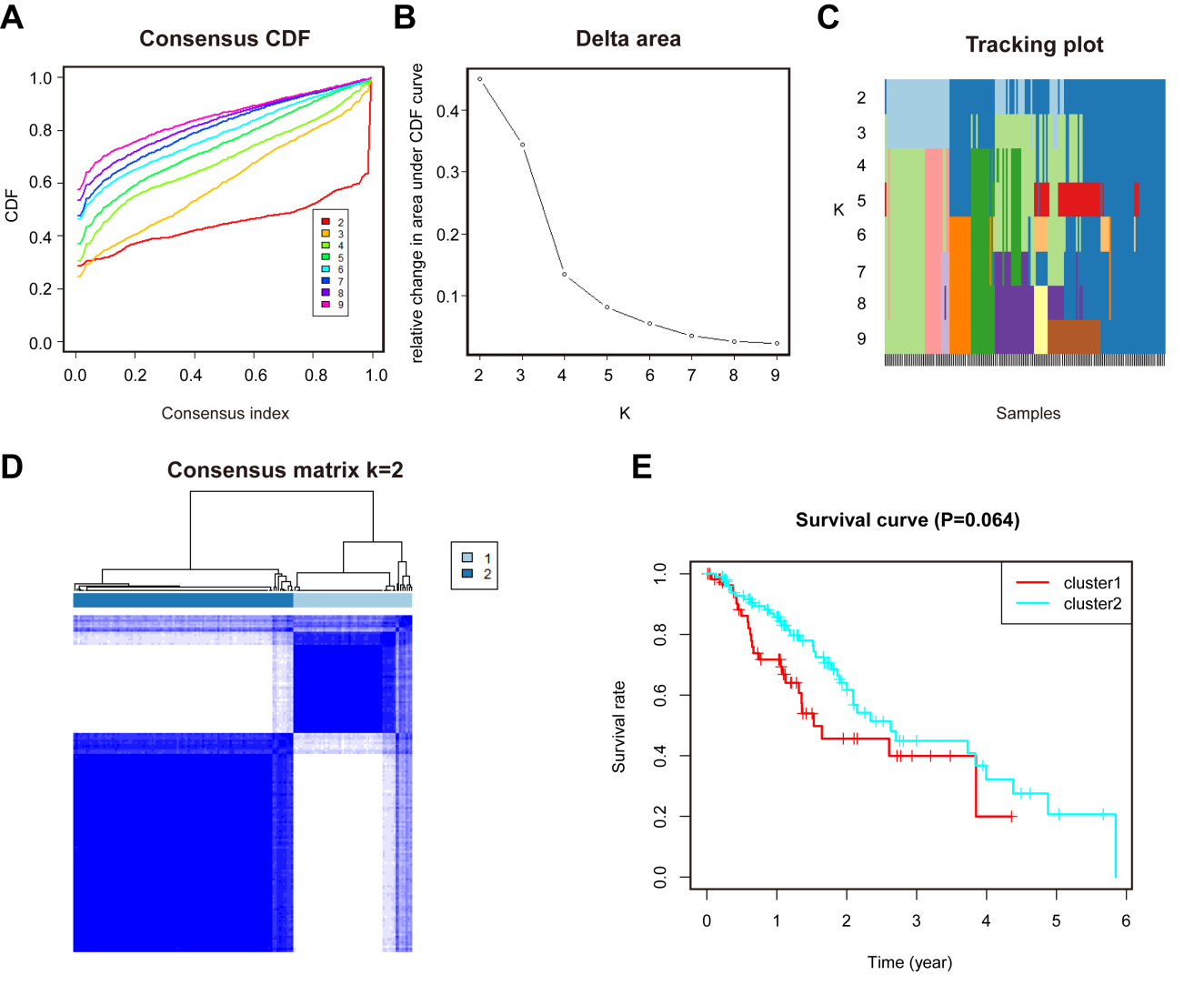


**Supplementary Figure 2. Identification of 2 clusters with different clinical outcomes in ESCA.** (A) Consensus clustering cumulative distribution function (CDF) for k = 2 to 9. (B) Relative change in area under CDF curve for k = 2 to 9. (C) The distribution of the sample when k is between 2 and 9. (D) Consensus clustering matrix for k = 2. (E) Kaplan-Meier OS curves for TCGA ESCA patients.


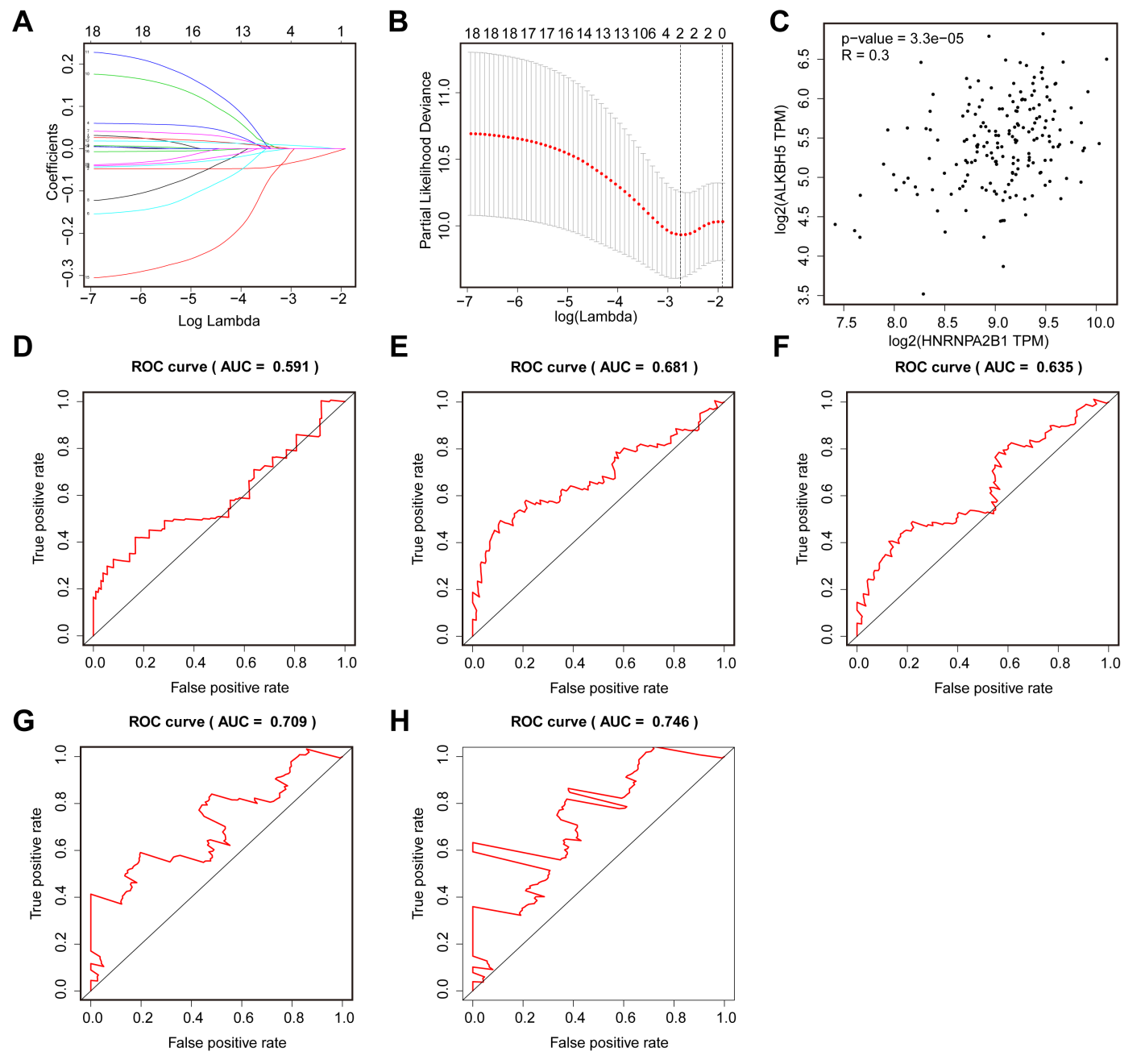


**Supplementary Figure 3. Identification of prognostic signature among m^6^A regulators.** (A, B) The coefficients are calculated by multivariate Cox regression using LASSO. The different colours in (A) indicated different 19 m^6^A related regulators. (C) The correlation of HNRNPA2B1 expression with ALKBH5 via online bioinformatics tool (<http://gepia.cancer-pku.cn/>) is analyzed. (D-H) ROC curves showed the predictive efficiency of the risk signature within 1 year (D), 2 year (E), 3 year (F), 4 year (G), and 5 year (H).


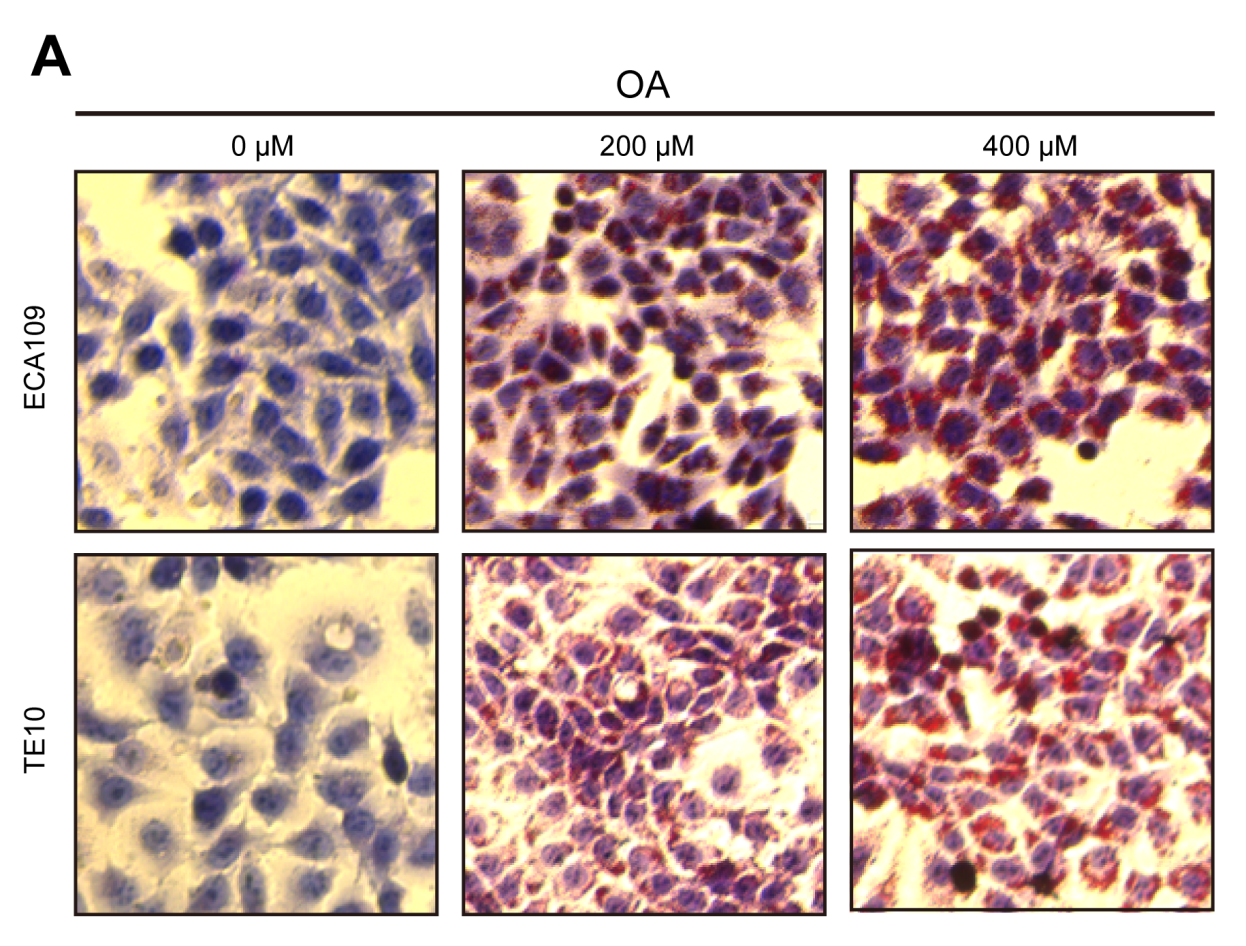


**Supplementary Figure 4. Representative figures of ESCC cells with OA.** (A) The ECA109 and TE10 ESCC cells were treated with OA at indicated dose for 24 h.

**
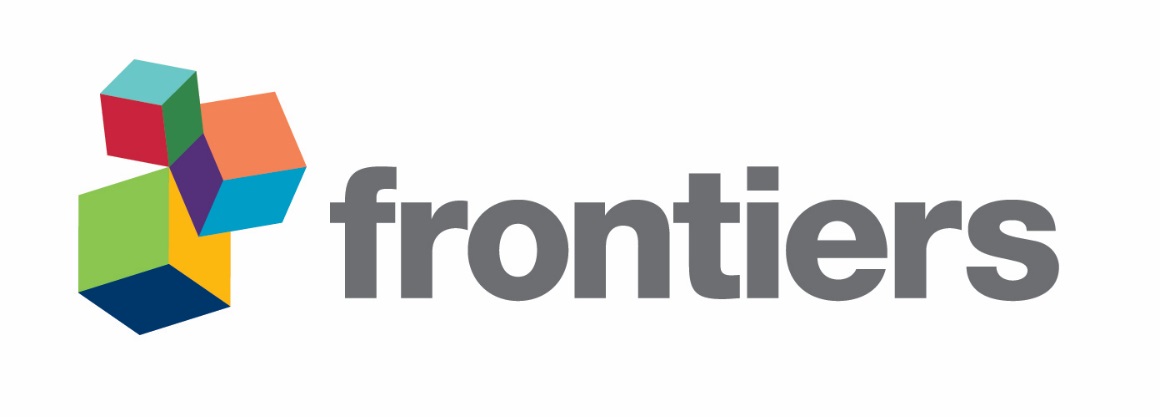
**
